# Baobab Laboratory Information Management System: Development of an Open-Source Laboratory Information Management System for Biobanking

**DOI:** 10.1089/bio.2017.0014

**Published:** 2017-04-01

**Authors:** Hocine Bendou, Lunga Sizani, Tim Reid, Carmen Swanepoel, Toluwaleke Ademuyiwa, Roxana Merino-Martinez, Heimo Meuller, Akin Abayomi, Alan Christoffels

**Affiliations:** ^1^ South African National Bioinformatics Institute, SA Medical Research Council Unit, University of the Western Cape, Bellville, South Africa.; ^2^ Bridging Biobanking and Biomolecular Research Across Europe and Africa (B3Africa) Consortium.; ^3^ National Health Laboratory Services, Tygerberg Hospital, Cape Town, South Africa.; ^4^ Division of Haematology, Department of Pathology, Faculty of Medicine and Health Sciences, Stellenbosch University, Tygerberg, South Africa.; ^5^ Human, Heredity and Health in Africa (H3Africa) Consortium.; ^6^ Medical Epidemiology and Biostatistics, Karolinska Institutet, Stockholm, Sweden.; ^7^ Institute of Pathology, Medical University, Graz, Austria.; ^8^ BBMRI-ERIC, Common Service IT, Graz, Austria.

**Keywords:** LIMS, biobank, open source, Plone, Baobab

## Abstract

A laboratory information management system (LIMS) is central to the informatics infrastructure that underlies biobanking activities. To date, a wide range of commercial and open-source LIMSs are available and the decision to opt for one LIMS over another is often influenced by the needs of the biobank clients and researchers, as well as available financial resources. The Baobab LIMS was developed by customizing the Bika LIMS software (www.bikalims.org) to meet the requirements of biobanking best practices. The need to implement biobank standard operation procedures as well as stimulate the use of standards for biobank data representation motivated the implementation of Baobab LIMS, an open-source LIMS for Biobanking. Baobab LIMS comprises modules for biospecimen kit assembly, shipping of biospecimen kits, storage management, analysis requests, reporting, and invoicing. The Baobab LIMS is based on the Plone web-content management framework. All the system requirements for Plone are applicable to Baobab LIMS, including the need for a server with at least 8 GB RAM and 120 GB hard disk space. Baobab LIMS is a server–client-based system, whereby the end user is able to access the system securely through the internet on a standard web browser, thereby eliminating the need for standalone installations on all machines.

## Introduction

Human biobanking refers to the collection, processing, and storage of biospecimens and the collection of associated demographic and clinical data for future research use. The extensive collections of biospecimens throughout Africa collected for either specific research, population studies, or part of normal diagnostics workup were not necessarily collected for prospective use by researchers and practitioners. As a result, such collections might not necessarily have followed or adhered to evolving bioethical paradigms and international biobanking best practices.^1,2^

However, the establishment of the concept of centralized biobanks across Africa through initiatives such as H3Africa, the AIDS Cancer Specimen Resource (ACSR), and the B3Africa (www.h3africa.org/consortium/projects, http://oham.cancer.gov/oham_research/programs/specimen_resource, www.b3africa.org) projects has highlighted the need for establishing and harmonizing national and regional biobank governance frameworks to address a relatively unregulated access to human and other ecological samples of academic interest in Africa. At the same time these governance frameworks fall in line with rapidly changing biobanking practices driven by modern technology.^3,4^ Similarly, a governance framework for IT infrastructure requirements that underlies a biobank does not exist.

According to the biological material tracking recommendations within the ISBER best practices, a computer-based inventory system should be in place to allow for the tracking and annotation of each incoming biospecimen into the biobank.^1^ A laboratory information management system (LIMS) is thus central to the informatics infrastructure that underlies biobanking activities. To date, a wide range of commercial and open-source LIMSs are available and the decision to opt for one LIMS over another is often influenced by the needs of the biobank clients and researchers, as well as available financial resources.

The National Health Laboratory Services (NHLS)—Stellenbosch University Biobank (NSB), a unit associated with the Division of Haematology at the Faculty of Medicine and Health Sciences, was established in 2012 initially through the ACSR project, and subsequently the NIH H3Africa funding initiative and required options for an LIMS implementation. The only option at the time was to consider a commercial LIMS because of time constraints to meet the growing need for biobanking services in South Africa. However, access to open-source LIMS software allowed us to consider a longer term implementation that would align with our sustainability plans. Bika LIMS^5^ and CaTissue (now evolved and known as OPENSPECIMEN^6^) were identified as long-term options based on input from active software developer and user communities.

The Bika LIMS, although not specific for human biospecimens, is part of the BIKA software ecosystem that includes a BIKA Health for healthcare laboratories and Bika Interlab for interlaboratory proficiency testing. Customization of the Bika LIMS software would provide the benefit of inheriting a range of electronic health record functions that are central to establishing a core facility to support personalized medicine research. The recently funded European project, B3Africa, was established to strengthen IT infrastructure and ethical governance frameworks that would bridge biobanking and biomedical research across Europe and Africa. This funding provided the impetus to revisit the biobanking IT infrastructure at the NSB and to accelerate the development of Baobab LIMS, an open-source LIMS for biobanking, as a strategy to provide a harmonized LIMS as an option for Africa.

A functional specification exercise^7^ in 2013 within the context of NSB biobanking requirements identified the following key modules as part of the extension to the existing Bika LIMS software, namely biospecimen kit assembly, shipping of biospecimen kits, storage management, analysis requests, reporting, and invoicing.

## Implementation

**FIG. 1.**
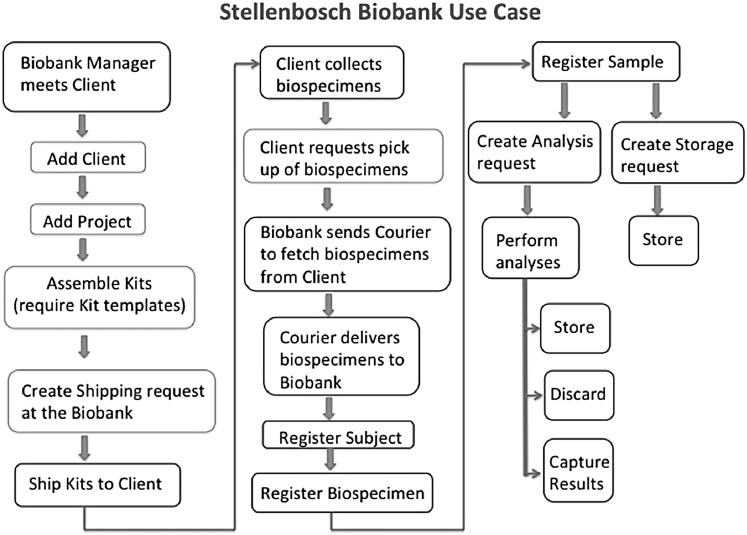
Flowchart of the activities within the NSB biobank. The activities within the NSB Biobank starting with a face-to-face meeting with a client to the final storage of the biospecimen and/or the analyses associated with that biospecimen.

Standard operating procedures (SOPs) associated with biological material inventory management were developed by the NSB team to inform LIMS workflow development. Other SOPs focusing on shipping, labeling, biospecimen procedures, and quality control were also developed in association with other H3Africa biobanks, and are publically available (http://h3africa.org/consortium/documents). The collection of SOPs underlies the NSB flowchart of biobank activities (Fig. 1) and subsequently the quality management system. Importantly, even with the use of electronic systems, it is important to keep hard copies of all documentation detailing the biospecimen passage from reception throughout storage to dissemination as a QC check.

## System Architecture

### Hardware and software

Baobab LIMS is based on the Plone web-content management framework (https://plone.org). All the requirements for Plone are applicable to the Baobab LIMS. As such, the Baobab LIMS is a server–client-based system, whereby the end user is able to access the system through the internet on a standard web browser, thereby eliminating disruptive workstation installations and inefficient software maintenance.

### Workflow description

The following processes and tasks were carried out by the NSB and the client to ensure that a biobank project is successfully implemented (Fig. 1):

(a) The biobank laboratory manager meets with one or more client representatives to decide on the requirements of a new biobank project. A client does not refer to a physical person but rather to an institution such as a laboratory, a hospital, or even another biobank that can be represented by one or more representatives. In the Baobab LIMS system, they are called “Client Contacts.” The project requires both parties to define the specimen types that will be collected and the biological analyses that are required at the biobank for all collected specimens. (b) The biobank prepares and assembles the kits that include equipment needed by the client to perform the sample collection, with one kit per participant. Mostly, a kit contains empty barcoded tubes and a form with a field for participant identifier, generated by the client. The collection forms are pre-barcoded with space for the client to use different barcodes for the tubes. In this way, the design of the forms minimizes the risk of assigning the incorrect tube to a participant. (c) The biobank ships the kits to the client by making use of external courier services. (d) The client receives the kits, collects the specimens in the associated tubes, and returns the kits to the biobank. (e) The biobank receives the kits from the client and registers the biospecimens in the system. (f) The biospecimens are then aliquoted for downstream analyses (the analyses defined in the project) or stored in freezers for later analysis.

### Workflow implementation

The Boabab LIMS modules were designed to suit most laboratory specimen reception workflows, with minimal customization and postinstallation. In the NSB biobank, these modules were adapted to suit the specific needs as set out in the SOPs that were developed. These SOPs described in detail the life cycle of a specimen as it moves through the laboratory, and which steps require specific documentation, such as the time the samples arrived in the laboratory, the time at which sample accessioning took place, which user was performing the analysis, the method used in the analysis request (linked to its own SOP), and the time of completion of the task, to name a few. In this way, the LIMS was able to adapt to almost all of the manual documentation processes that occur in this specific workflow.

Client and analysis request modules were imported from Bika LIMS into Baobab LIMS and customized to accommodate human biospecimen laboratory services. The following modules were custom built to support the routine activities of NSB (Fig. 1).

### Kit assembly

The biospecimen “Kit assembly” module provides the protocol needed to assemble kits that will be used to collect biospecimens in the field (Supplementary Data and Supplementary Figs. S1–S5; Supplementary Data are available online at www.liebertpub.com/bio). This module ensures that clients can order the appropriate kits for their biospecimen collection. The project that is registered at NSB will define the appropriate collection kit(s). For example, if a project will collect samples for DNA and RNA extraction at the client's laboratory, then the appropriate collection tubes with an assigned function and label must be shipped to the client for immediate implementation in the field or for storage under appropriate conditions until it is needed. The NSB maintains an inventory of kit components that can be put together and packaged for different sampling procedures and sample types depending on the client's need.

The kits include packaging material (styrofoam containers and corrugated shipping carton, absorbent material, and press lock biohazard plastic bag), gloves (optional), documentation (biospecimen submission form containing minimal information data, a shipping manifest (Supplementary Data and Supplementary Fig. S6), shipping checklist, shipping query form, and a workflow on how to pack and ship the kit), and the specific biospecimen collection tubes that are already labeled. For example, a kit required for DNA and RNA sampling might contain a 6 mL EDTA BD vacutainer or an 8.5 mL PaxGene Blood DNA tube and a 10 mL PaxGene Blood RNA tube or Tempus Blood RNA tubes depending on the downstream application. The kit assembly module provides the user the ability to select the appropriate kit template such as the templates for DNA or RNA sampling, which in turn is used to define the specific SOP required to assemble the material needed for the client's project. The kit template can be specific to one project or reused in different projects.

### Shipping

The shipping module ensures that the correct instructions are given to send the appropriate biospecimen containers (as packaged in the kits) to the client and subsequent e-mail notification to alert the client of incoming kits. Similarly, an e-mail notification based on the LIMS instructions will be sent to notify the NSB upon return of the kits from the client. The NSB, in consultation with the client, defines the appropriate containers to ship to the client and the shipping instructions to and from the client (Supplementary Data and Supplementary Figs. S6–S10). The kit, assembled in a size-appropriate box (Supplementary Data and Supplementary Figs. S4 and S5), includes a manifest that describes each kit and the barcodes (Supplementary Data and Supplementary Fig. S6).

### Storage management

Biospecimen, kits, and stock items are handled within the inventory management module. The inventory management module describes the steps needed for the storage location so that products can be ordered and stocks updated accordingly (Supplementary Data and Supplementary Figs. S10–S16). Biospecimen storage covers a hierarchy of storage levels: storage unit (or room), freezers, shelves, and boxes. The biospecimens are stored in cryoboxes in various sizes depending on the size of the collection tube and cryotubes. For the other storage types (kits and stocks), there is no hierarchy to respect and the positions can be created at any level.

### Freezer management

Different kinds of storage for biospecimens and aliquots exist inside the NSB depending on biospecimen type as well as the need for short- or long-term storage. The freezer management module describes the steps needed to define the structure that matches the physical storage in the NSB: rooms (within rooms), freezers, shelves, cryoboxes, and positions/locations (Supplementary Data and Supplementary Figs. S17–S20). A freezer contains shelves that contain cryoboxes. The last storage level at the NSB, namely cryoboxes, contains the positions reserved for biospecimens and aliquots. Similarly, the liquid nitrogen freezer and/or dewar contain, respectively, racks that contain canes. The box and/or cane can have multiple positions for biospecimen storage. Three classes (content types) were used to design the freezer management module (Supplementary Data and Supplementary Figs. S18 and S21), namely storage unit (room), storage level (freezer, shelf, and cryobox), and storage location (positions inside cryobox). This class inheritance was implemented using the object database ZODB (www.zodb.org/en/latest).

### Security and administration management

Plone (the web-content management platform for Bika software) is based on the Zope framework. Zope provides built-in security functionality that allows us to define roles with permissions. A “permission” controls whether logged-in or anonymous users with a specific role can execute code and access contents. Each NSB staff member and client will have specific assigned roles and a level of security. Baobab LIMS also inherits Plone's secure version-controlled document management system and reviewer workflows and audit trails.

Plone uses ZODB to store user data. ZODB, compared with SQL-based databases, is not vulnerable to injection as it uses binary format that cannot have user data inserted. Plone authenticates users in its own database using an salted secure hash algorithm (SSHA) hash of their password. Using its modular authentication system, Plone can also authenticate users against common authentication systems such as LDAP and SQL as well as any other system for which a plugin is available (Gmail, OpenID, etc.). After authentication, Plone creates a session using an SHA-256 hash of a secret stored on the server, the *userid*, and the current time. This is based on the Apache auth_tkt cookie format, but with a more secure hash function.

### Analysis request

A global list of available analyses is defined by the capabilities of the biobank laboratory. At the project level, analyses are defined for biospecimens based on the requirements of the project, such as DNA and/or RNA extraction applied to blood samples, and the resulting quality and purity results of the extracted DNA and/or RNA. Results of these analyses are registered and reported to the client (Supplementary Figs. S22 and S23). The data are imported into Baobab LIMS through an instrument interface such as the biodrop interface (Supplementary Data and Supplementary Fig. S24).

### Availability of software

Source code is available directly from (https://github.com/hocinebendou/baobab.lims.git) or (http://christoffels.sanbi.ac.za/index.php/software/software-downloads).

A demo site has been configured at b3abiobank.sanbi.ac.za/demo (login = admin; password = admin).

## Discussion

High-throughput genetic tools allow researchers to rapidly analyze thousands of biospecimens, as is common in consortia focused on population-based studies. In response, biobanks have to carefully consider the appropriate IT infrastructure that can meet the demands of large genetics studies. The Baobab LIMS was designed as an open-source alternative for use in a resource-limited setting to meet the demands of increasing biospecimen collection.

During the development of Baobab LIMS, a new open-source LIMS, Acquire, was published with a focus on pathology biospecimens.^8^ This tool integrates the inventory management functionality of OpenSpecimen with modules specifically designed to meet requirements of researchers in a pathology laboratory. However, tools such as OpenSpecimen do not handle the specific biobank activities of NSB, which includes supporting prepackaging of biospecimen laboratory kits for clients (Fig. 1).

The establishment of centralized biobanks in Africa has drawn attention to the issues of interoperability between biobanks and harmonization of terminology used by each biobank. These concerns are not unique to African biobanks.^9,10^ Data standards such as MIABIS^11^ provide an ideal platform to integrate data among biobanks. The advantage of using the same terminology across biobanks was demonstrated through the development of the virtual Breast Cancer Campaign Tissue Bank.^12,13^ Although a biobank catalogue is not currently available at NSB, we envisage a simplified application programming interface (API) that would allow users to access summary information using data stored in Baobab LIMS.

Qinlan et al.^13^ suggested an increased role of ethical boards, governance, accreditation bodies, and funders to ensure that groups being authorized to collect samples have sufficient informatics capabilities to ensure the samples are used. This suggestion ensures that teams authorized to collect samples will also have the technical skills to ensure that the associated data are managed correctly. This concept relates to issues of biobank sustainability. Technical costs at a biobank go beyond supporting an LIMS and instead should incorporate a biobanking informatics management system that allows the biobank the flexibility to meet the growing technical demands of making data available to a wider scientific community beyond biospecimen handling in a laboratory. In this context, and doubling the cost suggested by Dowst et al.^8^ for maintaining their Acquire LIMS, the cost of employing a linux administrator (20% full-time equivalent [FTE]), database administrator (10% FTE) and a computer programmer (25% FTE) in a South African setting would cost 30,000 U.S. dollars. The demands placed on IT staff require that biobanks have dedicated staff for these technical roles, thereby increasing personnel costs at least fivefold. These costs are a key consideration for centralizing and harmonizing biobanking in Africa. Unfortunately, local, regional, and international funders need to appreciate the importance of this critical component required for modern biobanking and the academic advantage of interoperability across evolving biobanks.

The adoption of Baobab LIMS by a wider user community will require more generic and configurable workflows. Nevertheless, Baobab LIMS has become a central component in the eB3Kit of the B3Africa H2020 project and is also included in the BBMRI-ERIC software catalogue, and in the open BIBBOX application store.^14^

## Supplementary Material

Supplemental data
