# Supplementary material for: Baobab Laboratory Information Management System: Development of an Open-Source Laboratory Information Management System for Biobanking
Source: Biopreserv Biobank. 2017 Apr 1;15(2):116–20. doi: 10.1089/bio.2017.0014 (PMC5397207; doi:10.1089/bio.2017.0014)
Supplement: Supplemental data [file Supp_Data.pdf]

Supplementary Data

This document describes (with screenshots) each of the modules that were developed to produce the Baobab Laboratory Information Management System (LIMS) namely (1) kit assembly, (2) shipping, (3) storage management, (4) inventory management, (5) freezer management, (6) sample storage management, (7) biospecimen registration, and (8) analysis request (AR) by a client.

Kit Assembly

The following kit attributes are maintained with a kit template:

- Kit name
- Kit type/class
- Labeling requirements
- Assembly SOP
- Component list (select from components available in the database)
- Packaging outline
- User instructions on packaging of kits, shipping and completion of compulsory forms.
- Temperature monitors depending on sample type and shipment type.

Other required regulatory documentation that accompanies all shipments may be loaded to the LIMS before shipments or is e-mailed to the receiving laboratory or biobank. These include the ethic approval documentation; the biospecimens deposit material transfer agreement and permits. Submission sites also have to notify the receiving laboratory and biobank of incoming shipments and prepare the following required forms before shipment. A shipment checklist is completed by the submission site and is for internal use only. The shipment manifest/notification and the shipment receipt confirmation and query form should be sent by e-mail to the receiving site at the time of shipment. The courier's waybill number and copies of commercial invoice and permits must also be sent with the shipment.

Kit assembly

The submission sites (the biobank's clients) order kits from the biobank based on a particular project to be carried out on a specific case study. For example, blood samples are to be collected to carry out DNA extraction and subsequent analysis on a group of participants. The laboratory manager navigates to ''Kit assembly'' form and selects from a list of prepacked/designed kit templates the template that is mostly used in the field by the client. Many variations of kit templates can be created for the DNA blood sampling kit based on the collection tubes as defined for specific downstream applications. If the appropriate kit template is not available, then the laboratory manager can create the desired kit with the appropriate collection tubes. The kit template consists of a list of components required by the client for sample collection and subsequent shipment. Supplementary Figure S1 shows a DNA blood sampling kit template with two components: one pair of gloves and two blood tubes.

The kit template is used to avoid the recurring selection of components during kit assembly. The kit template that consists of components is imported once for every number of kits to be assembled. In the kit assembly form (Supplementary Fig. S3), the selected kit template will define the components and their quantities that must be added to each kit. The biobank staff member will select the specific kit template from a drop-down menu (Supplementary Fig. S3) and the total number of kits that must be prepared (based on the client's requirements).

The kit assembly form specifies which stored stock items are to be used for the assembly of the kit from the list of stock items that underlies the inventory management system. The number of consumables that are required for the kit assembly is tracked and audited within the inventory management system. Biobank staff can follow this audit trail to know when consumables are running low and need to be restocked.

The biobank staff members store the assembled kits in the corresponding storage under the correct conditions until the kits are shipped to the client (Supplementary Fig. S2).

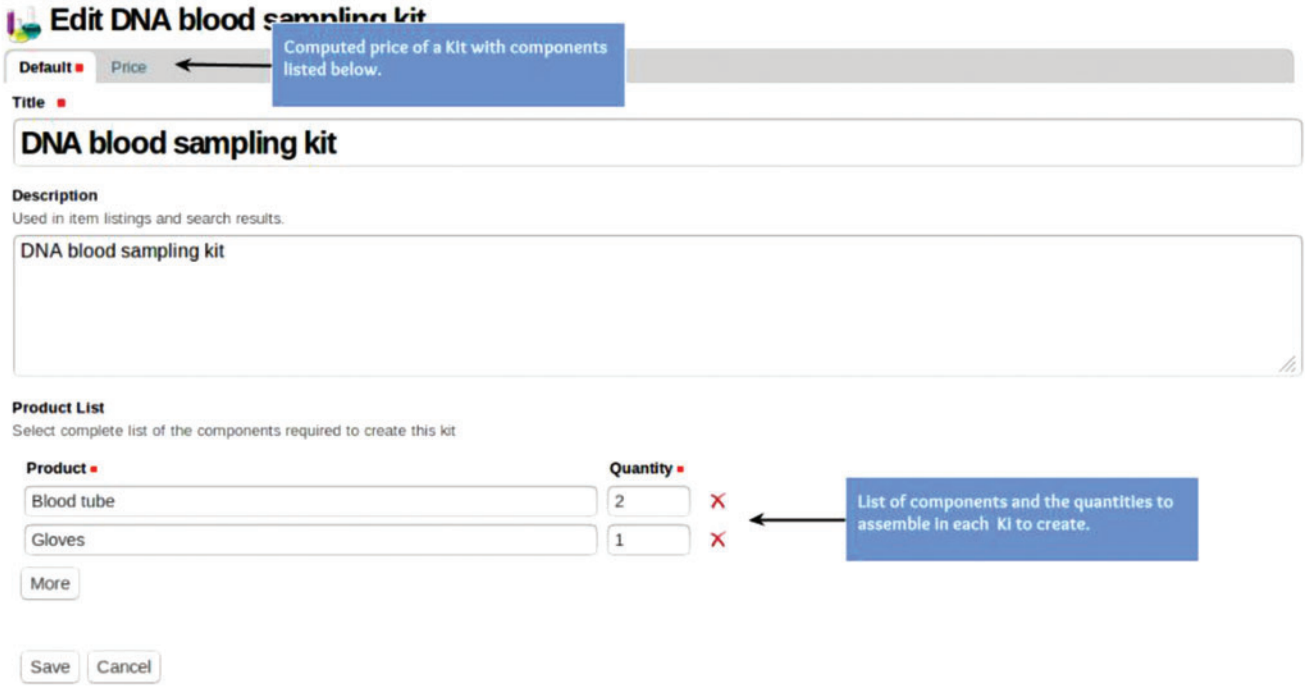

SUPPLEMENTARY FIG. S1. Creation of a DNA blood sampling kit.

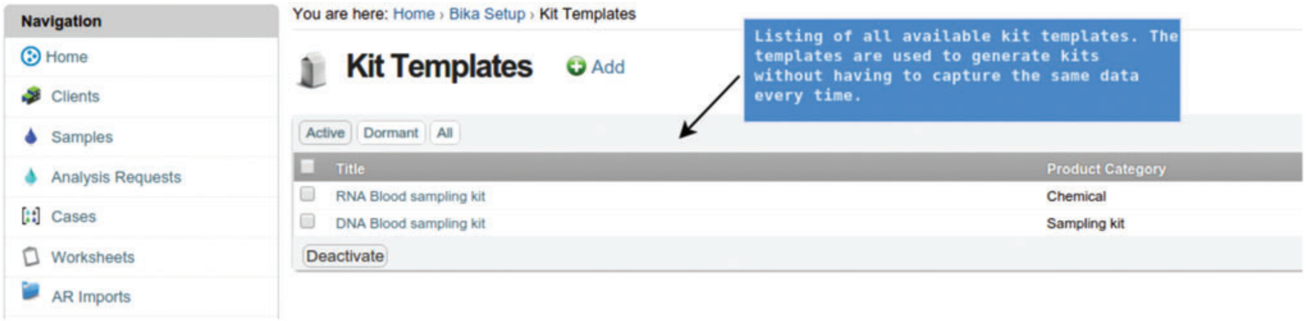

SUPPLEMENTARY FIG. S2. The list of kits available in BikaBiobank LIMS. LIMS, Laboratory Information Management System.

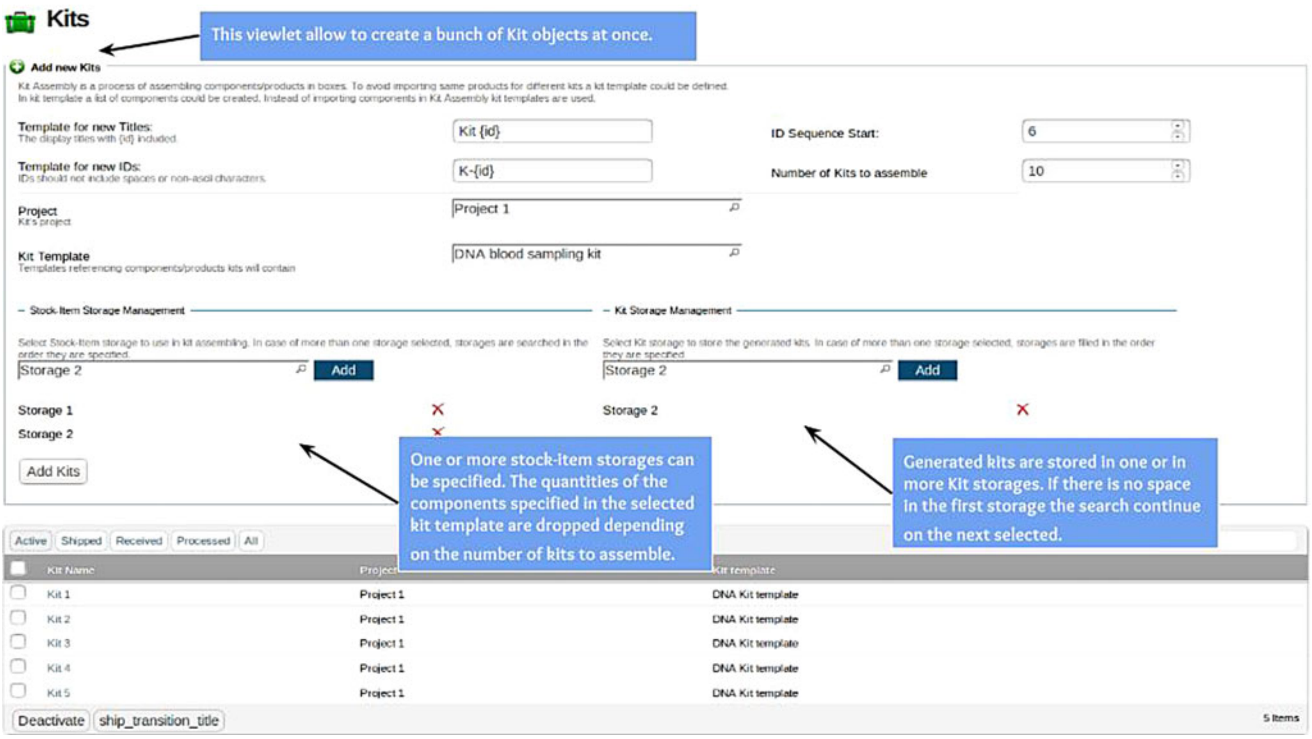

SUPPLEMENTARY FIG. S3. Kits creation. The specific kit template (DNA blood sampling kit) is selected and other details are added. This information is then ready for someone to physically collect materials from the storage room. Consumables are scanned to tell inventory system that a number of consumables have been released for kit assembly.

Shipping

The Biobank in consultation with the client defines the appropriate containers to ship to the client. The kits are assembled as per Kit assembly section. In our example hereunder, one kit was prepared that contains one predesigned barcoded acid citrate dextrose collection tube with an assigned function associated with the label in a size-appropriate styrofoam box (Supplementary Fig. S4). The collection tubes are secured with laboratory tape with the barcoded label facing down. Absorbent material is placed within the cavity of the box and covers all components. A lid is added and waterproof tape used to seal the lid to the body of the box. The sealed styrofoam box is placed in a press-lock plastic bag The plastic sealed kit is placed within a corrugated shipping carton box (Supplementary Fig. S5) with an associated manifest (Supplementray Fig. S6) in the pockets of the plastic bags. The courier waybill, commercial invoices, and permits if applicable are placed on the outside of courier box not covering the markings on the box. The shipping notification/manifest (Supplementary Fig.6) and the confirmation and query forms are sent to the biobank to notify them of an incoming shipment.

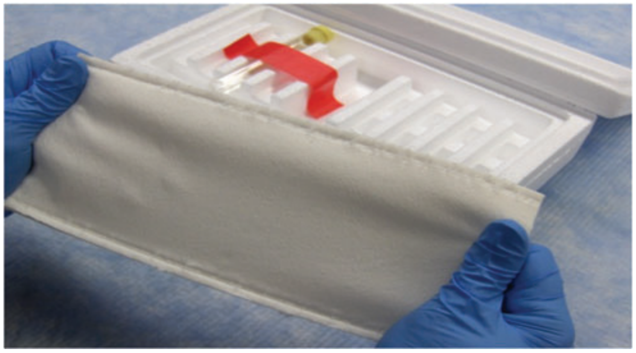

SUPPLEMENTARY FIG. S4. A kit comprising collection tube and the associated components prepared for one patient or individual.

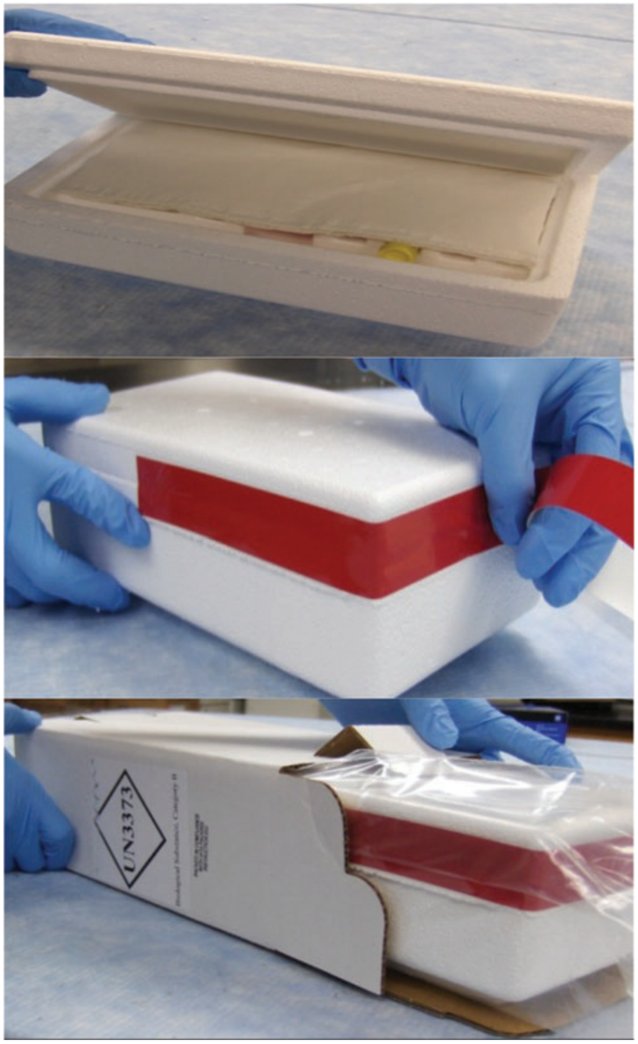

SUPPLEMENTARY FIG. S5. Kit assembly instructions show packaging of kit according to IATA standards and assembly into a corrugated shipping box with the correct markings.

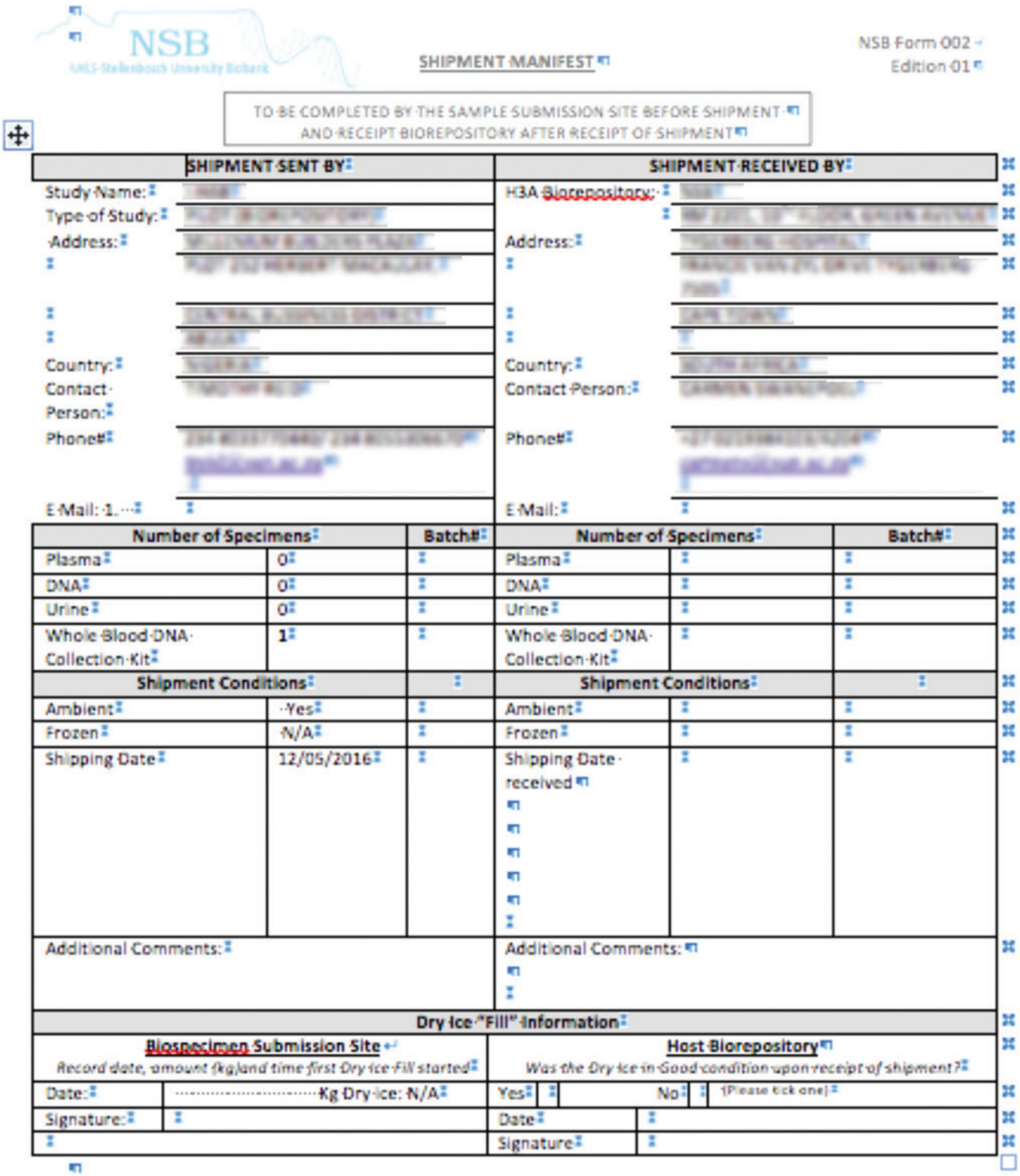

NSB
NHLS-Stellenbosch University Biobank

SHIPMENT MANIFEST

NSB Form 002
Edition 01

TO BE COMPLETED BY THE SAMPLE SUBMISSION SITE BEFORE SHIPMENT AND RECEIPT BIOREPOSITORY AFTER RECEIPT OF SHIPMENT

SHIPMENT SENT BY		SHIPMENT RECEIVED BY	
Study Name:	[illegible]	H3A Biorepository:	[illegible]
Type of Study:	[illegible]		[illegible]
Address:	[illegible]	Address:	[illegible]
	[illegible]		[illegible]
	[illegible]		[illegible]
	[illegible]		[illegible]
Country:	[illegible]	Country:	[illegible]
Contact Person:	[illegible]	Contact Person:	[illegible]
Phone#	[illegible]	Phone#	[illegible]
E-Mail: 1.		E-Mail:	

Number of Specimens		Batch#	Number of Specimens		Batch#
Plasma	0		Plasma		
DNA	0		DNA		
Urine	0		Urine		
Whole Blood DNA Collection Kit	1		Whole Blood DNA Collection Kit		
**Shipment Conditions**			**Shipment Conditions**		
Ambient	Yes		Ambient		
Frozen	N/A		Frozen		
Shipping Date	12/05/2016		Shipping Date received		
Additional Comments:			Additional Comments:		

Dry Ice "Fill" Information				
**Biospecimen Submission Site** *Record date, amount (kg) and time first Dry Ice Fill started*		**Host Biorepository** *Was the Dry Ice in Good condition upon receipt of shipment?*		
Date:	............Kg Dry Ice: N/A	Yes	No	(Please tick one)
Signature:		Date		
		Signature		

SUPPLEMENTARY FIG. S6. A shipping manifest. This form is included in the assembled kits. The client completes this form before shipping the biospecimens to the biobank.

Shipping instructions from the biobank

The ‘‘shipments’’ page (Supplementary Fig. S7) shows the list of shipment instructions including those that are pending (see tab in top lend hand corner). The ‘‘add’’ button (Supplementary Fig. S7) pops up a new window (Supplementary Fig. S8) with a form that captures all the instructions to ship an assembled kit to the client. The fields in this form include the details of the courier company, a Kit-ID (defined for the assembled kit) (Supplementary Fig. S9), the date of shipping to the

client, the person giving the shipping instructions, and to whom the kits will be shipped. After the request, the biobank calls the courier company to make arrangements for them to come and fetch the assembled kits.

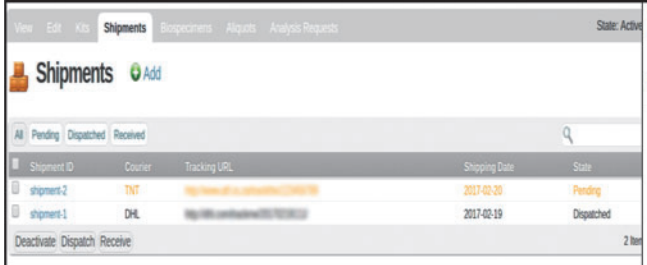

SUPPLEMENTARY FIG. S7. Summary of shipments. The "add" button (Black box) provides a new form (Supplementary Fig. S9) to activate the shipping instructions for an assembled kit.

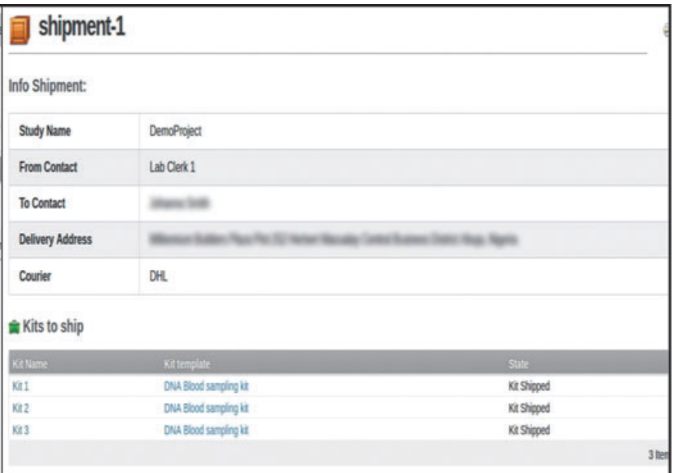

SUPPLEMENTARY FIG. S8. Shipping information. The assembled kits are specified in this shipping form together with the details of the courier. This form must be completed before calling the courier company.

Shipping instructions from the client

The assembled kits are delivered to the client and biospecimens (blood, urine etc.) are collected in prelabeled tubes according to the kits that were assembled (i.e., one kit per patient). The client logs into the LIMS system and uses the shipping module to inform the biobank that the kits are ready for collection. An e-mail is automatically generated and the biobank is notified that the incoming kits are to be expected. The biobank contacts the courier to collect the biospecimens from the client or the shipper calls the courier as the shipment has been prearranged by the biobank.

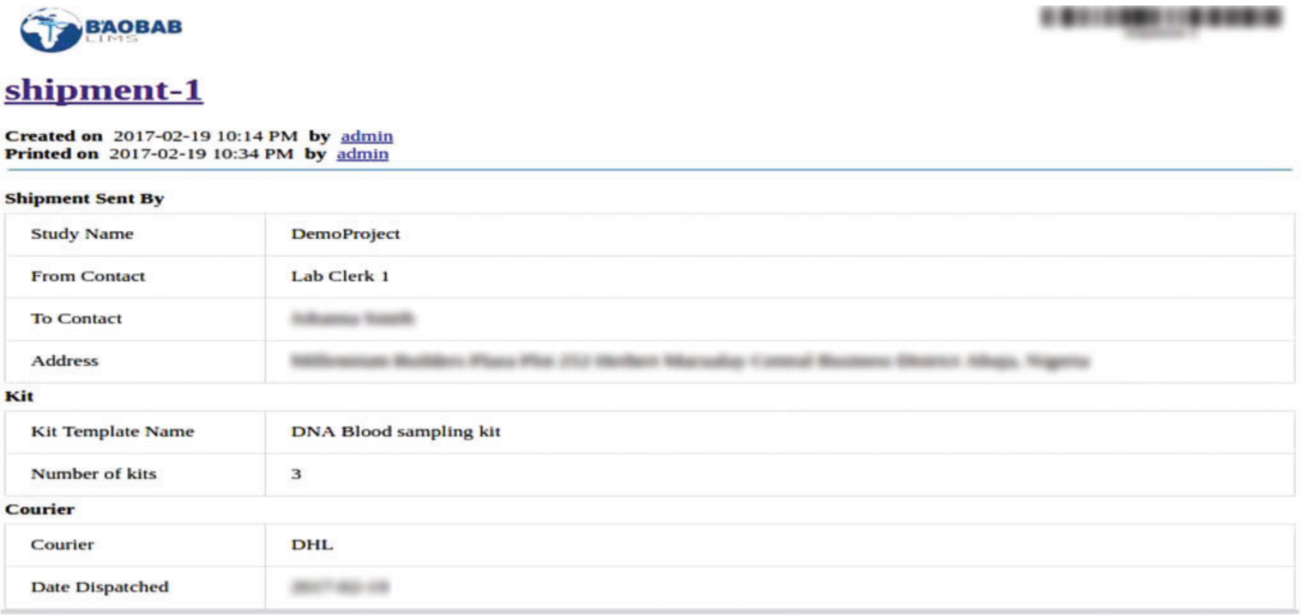

SUPPLEMENTARY FIG. S9. Summary window for a shipping instruction. The summary shown in this figure reflects the shipping instructions as defined in Supplementary Figure S5. This information is for record keeping and represents the information needed for the couriers to collect the biospecimens.

Storage Management

Supplementary Figure S10 shows the form for storage creation. The form contains three tabs: storage units, managed storage, and unmanaged storage.

Storage units

These sections are used for creating the structure that matches the physical storage. The storage unit tab can contain multiple storage units as well as managed or unmanaged storage, but items cannot be stored directly in storage units. Storage units are defined as ''room,'' ''freezer,'' and ''shelf'' (Supplementary Fig. S10).

Managed storage

This section contains a set number of positions for storing objects, for example, boxes that can store 36 tubes each, or shelves that can store stock items. Once all positions are occupied, the storage itself will be flagged as occupied, and when a position becomes available, the storage becomes available too. Items can be stored in specific positions, or the storage itself can be selected, in which case a position is chosen automatically—useful for storage of bulk items (Supplementary Fig. S11).

Unmanaged storage

This section does not restrict the number of items that can be stored. These storage units will be available for selection until they are manually flagged as occupied (Supplementary Fig. S12).

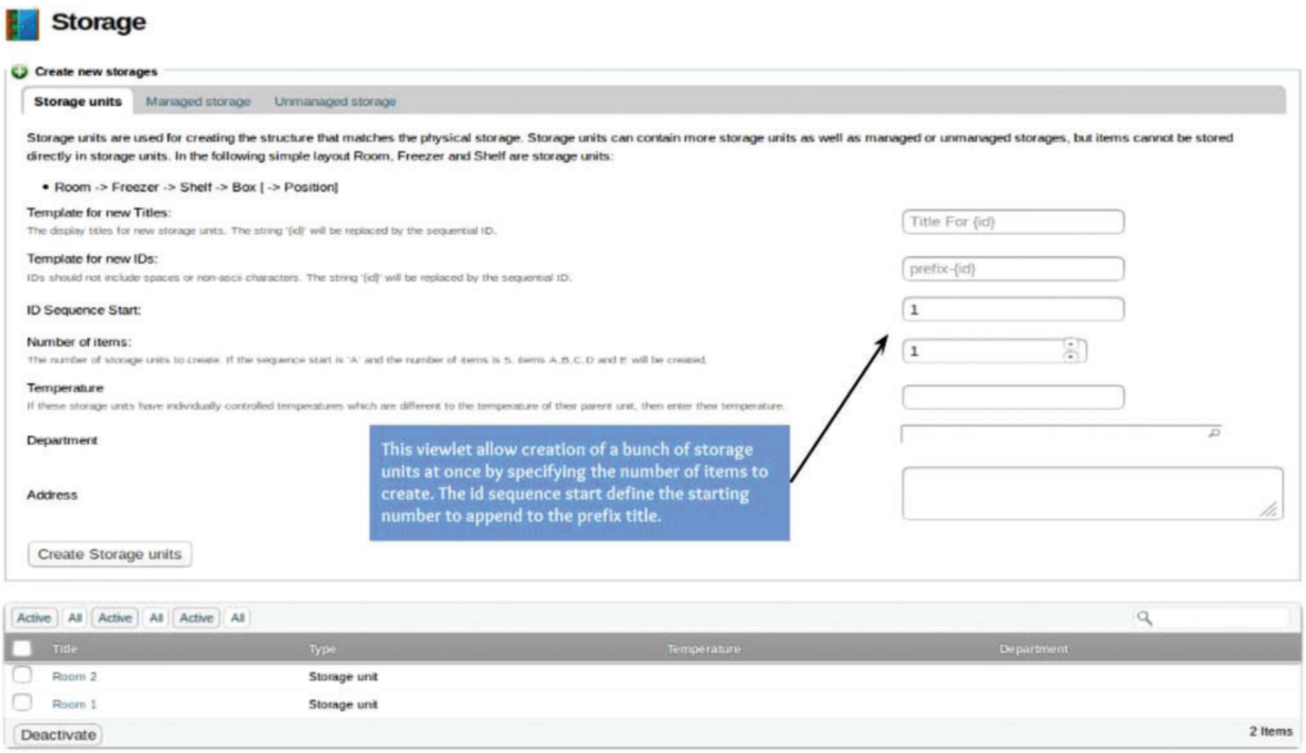

SUPPLEMENTARY FIG. S10. Form for creating storage units.

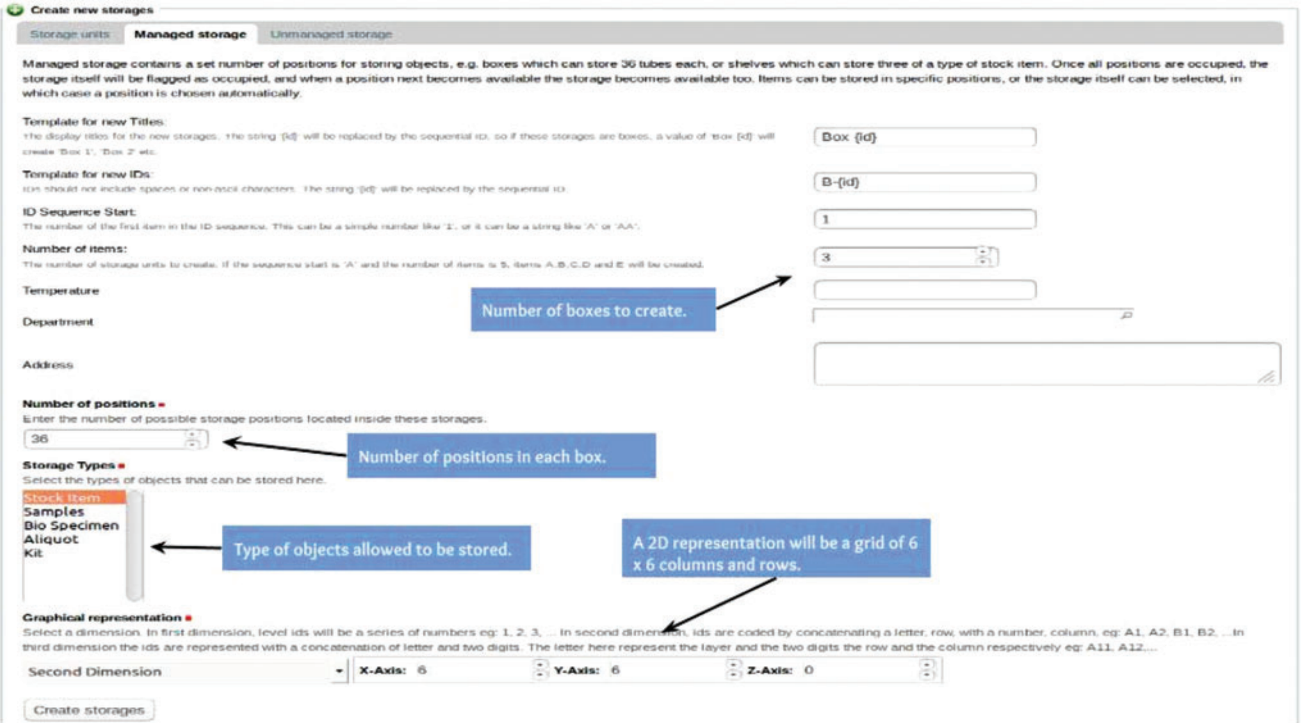

SUPPLEMENTARY FIG. S11. Storage management. The number of positions in each storage must be set.

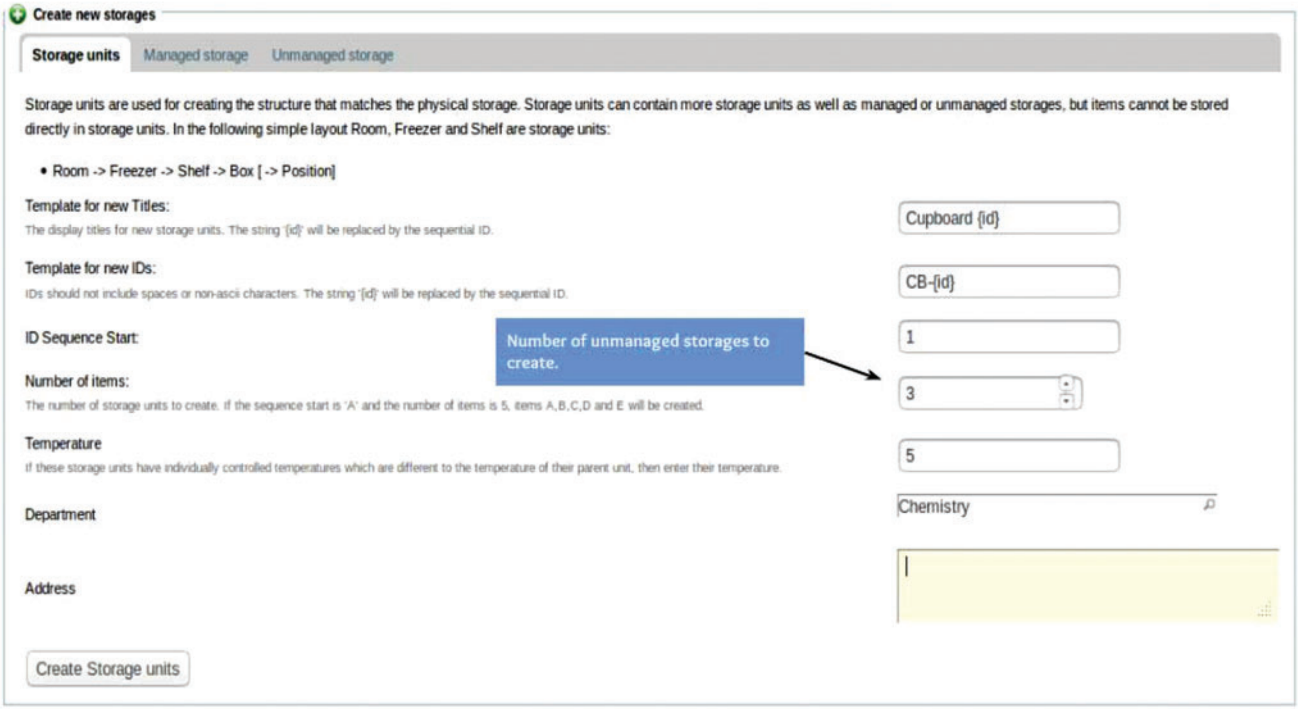

SUPPLEMENTARY FIG. S12. Unmanaged storage. No need for specifying the number of positions. The storage can only be manually set to ‘‘fully occupied.’’

Inventory Management

Stock items (products) and kit components can be stored in managed or unmanaged storage. Supplementary Figure S13 shows an example of managed storage created for storing stock items. When assigning storage locations for the first time, the storage positions are all free and are shown in green in the layout. Attributing positions for ordered products will change the positions to occupied and are shown in red (see next section).

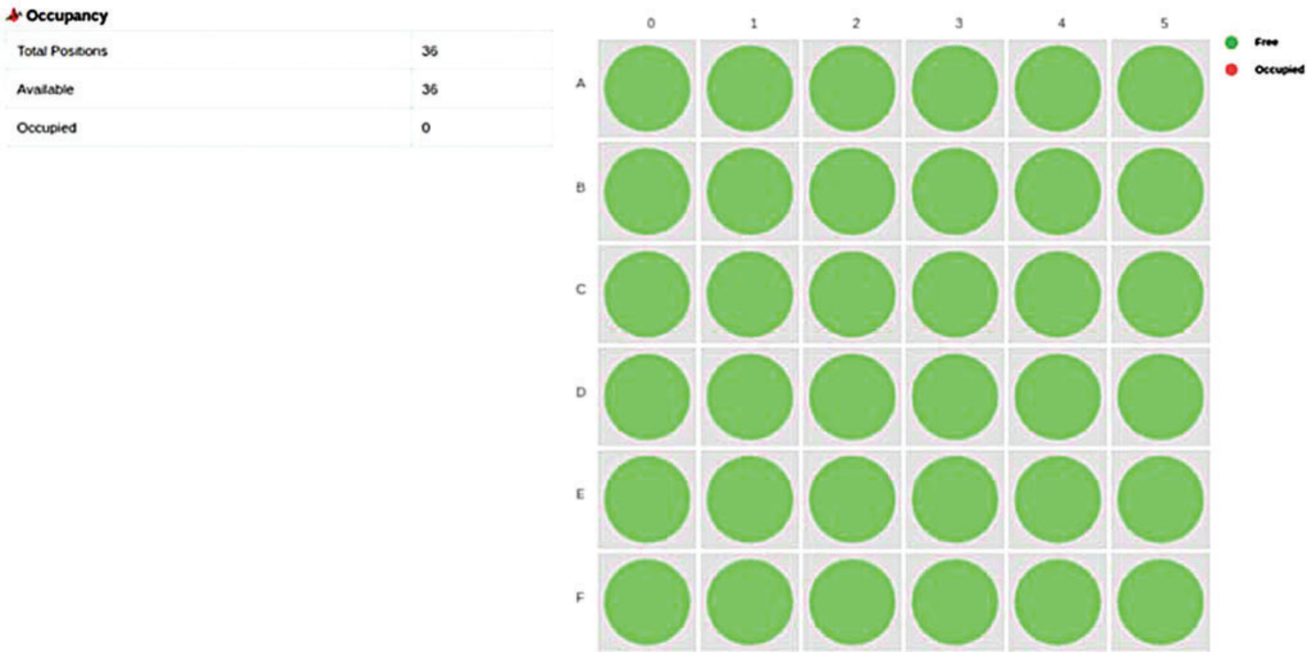

SUPPLEMENTARY FIG. S13. Graphical representation of a storage with 36 positions.

Stock orders from suppliers

Stock and products should be provided before a kit is created and assembled. Specific products are ordered from a supplier. Supplementary Figure S14 shows the list of products that are available for a supplier called ‘‘Instruments Inc.’’ An order is placed for five quantities of ‘‘blood tubes’’ and three quantities of ‘‘pipette’’ and depicted in Supplementary Figure S15.

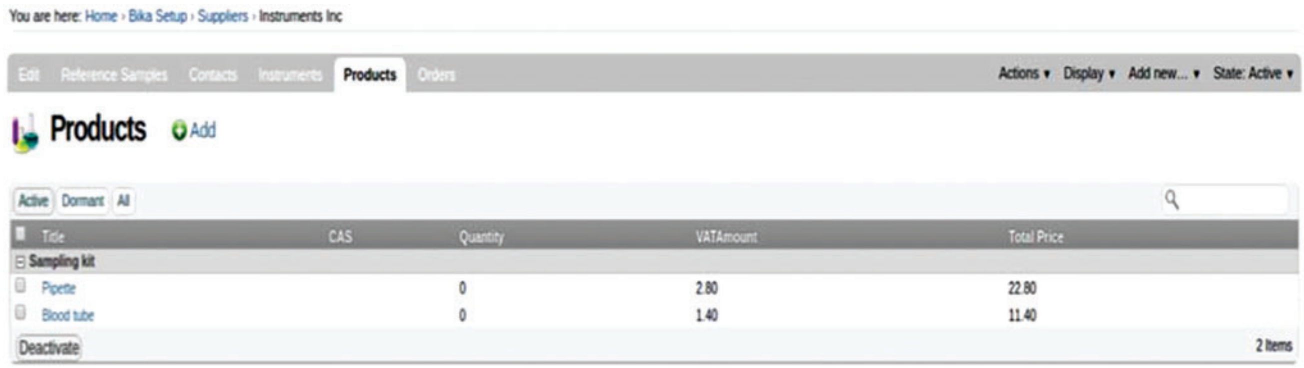

SUPPLEMENTARY FIG. S14. Products available for a supplier.

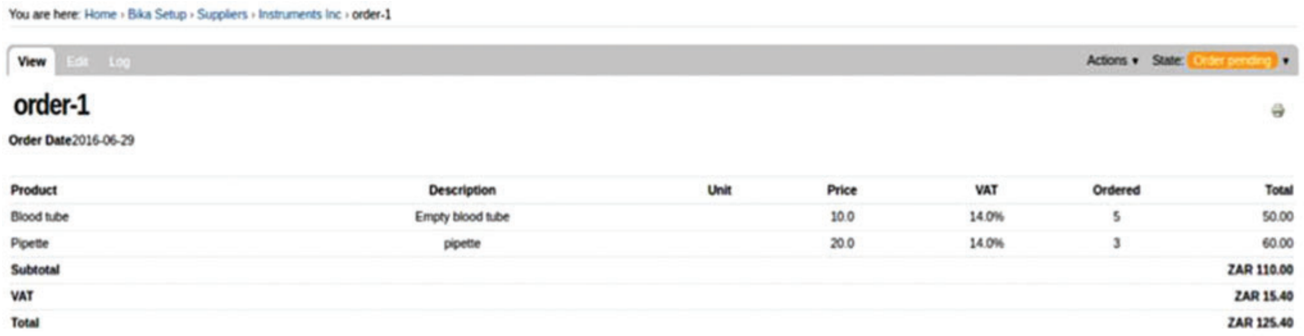

SUPPLEMENTARY FIG. S15. An order submitted to supplier (Instruments, Inc.)

Create stock items for storages

The products are automatically created as stock items after they are received from the suppliers. At this point, the stock items are ready for storage in the location defined in the next form (Supplementary Fig. S16).

Supplementary Figure S16 shows the precedent order when products are received. In that state, the user will be able to select the quantity and the storage location. There are scenarios where the quantities received are more than the available positions in the location selected. The system will only store the number of stock items equivalent to the number of available positions. Note that the user can select other locations if they exist. The order will be on state Stored only when all stock items are stored.

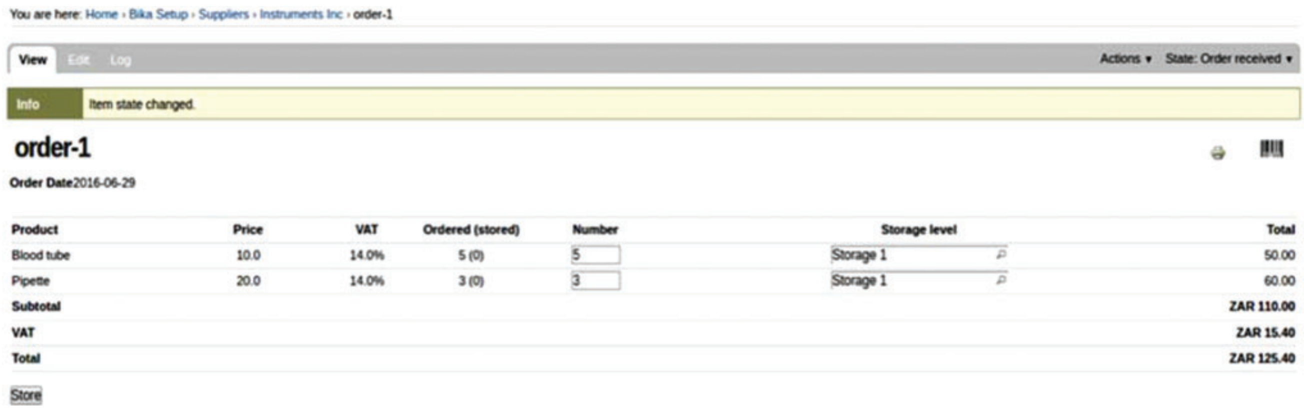

SUPPLEMENTARY FIG. S16. The order in which stock items are stored.

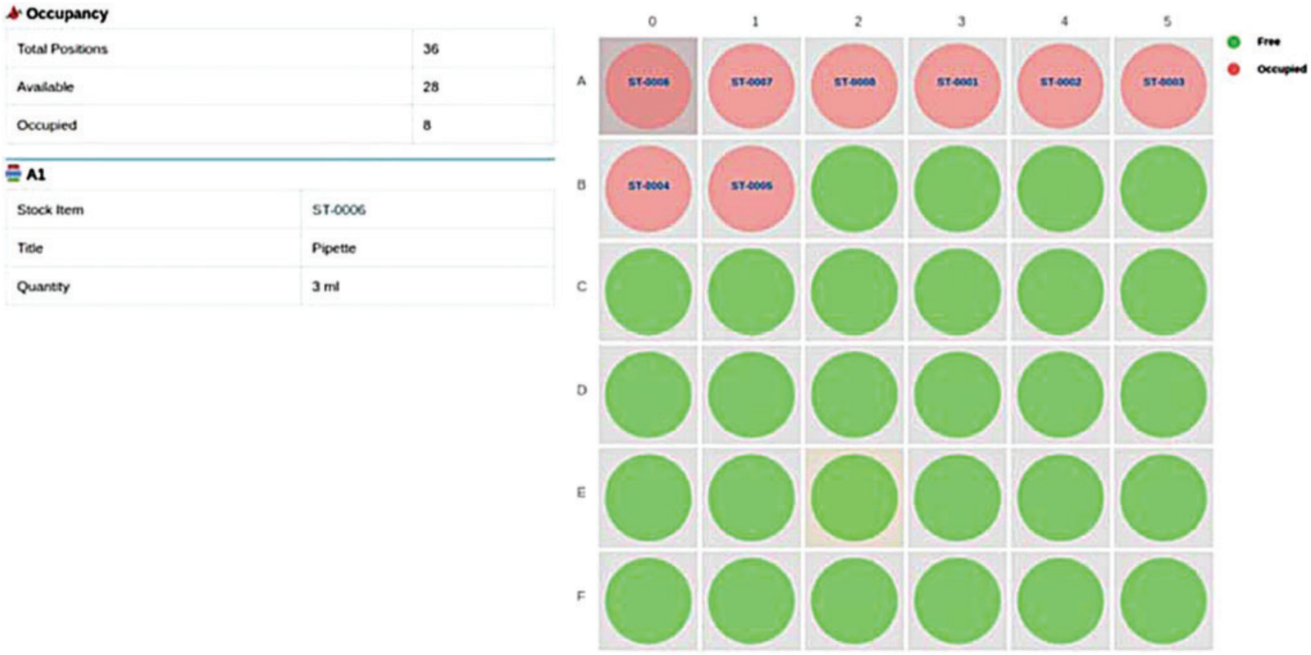

SUPPLEMENTARY FIG. S17. Storage after ordering.

Freezer Management

In contrast to inventory management, freezer management follows a certain structure and order for creation. This order can be obtained only with using managed storage (Supplementary Fig. S11).

Storage position engine

Three classes (content types) were used to design the freezer management module (Supplementary Fig. S18) namely: Storage Unit (room), Storage Level (freezer, shelf, and box), and Storage Location (positions inside box).

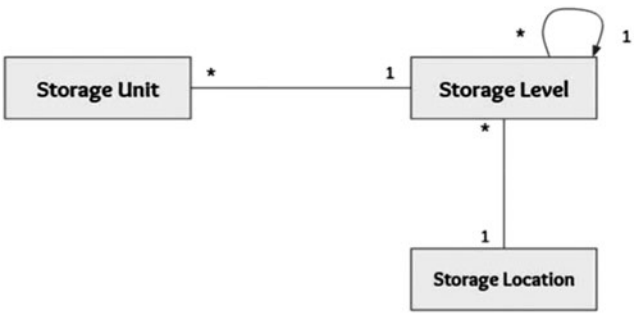

SUPPLEMENTARY FIG. S18. Freezer management relational diagram. Depicted in this diagram are Storage Unit: room; Storage Level: freezer, shelf, and box; and Storage Location: positions inside box.

Use case, freezer configuration. Plone and Zope frameworks use ZODB, an object database for storing records (objects). Objects, by following class inheritance concept, could be represented as a tree whereby a given object should have a parent. Supplementary Figure S19 shows an example for how storage is represented.

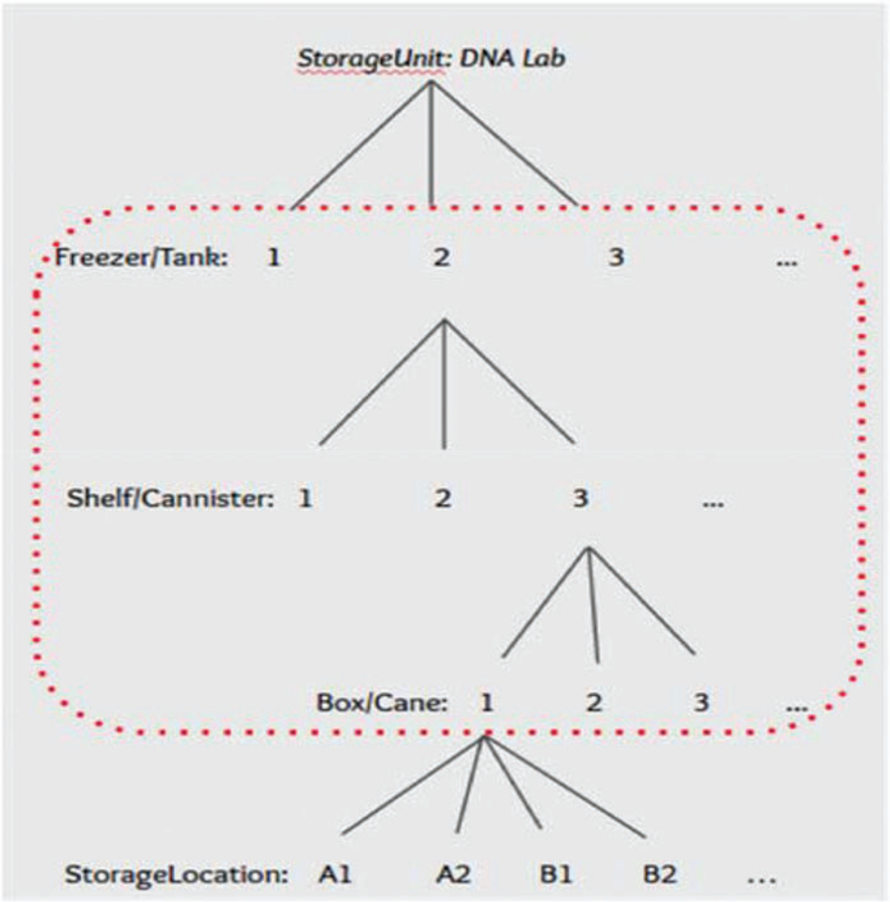

SUPPLEMENTARY FIG. S19. Freezer storage tree representation.

Storage configuration

For both sample storage and inventory management, positions are set up once for every freezer, cupboard, or room, during system configuration, and only again when new freezers arrive or older freezers are decommissioned.

Sample Storage Management

Following the structure described in the precedent section, samples that can be biospecimens or aliquots are stored in position within boxes created using ‘‘Managed storage’’ form (Supplementary Fig. S11).

Graphical representation

The different storage positions for samples are graphically depicted in Supplementary Figure S20. Each circle represents an object position. A state of a position could be ‘‘free,’’ ‘‘reserved,’’ or ‘‘occupied.’’ A circle with a different color represents each state: free = green; reserved = blue, and occupied = red.

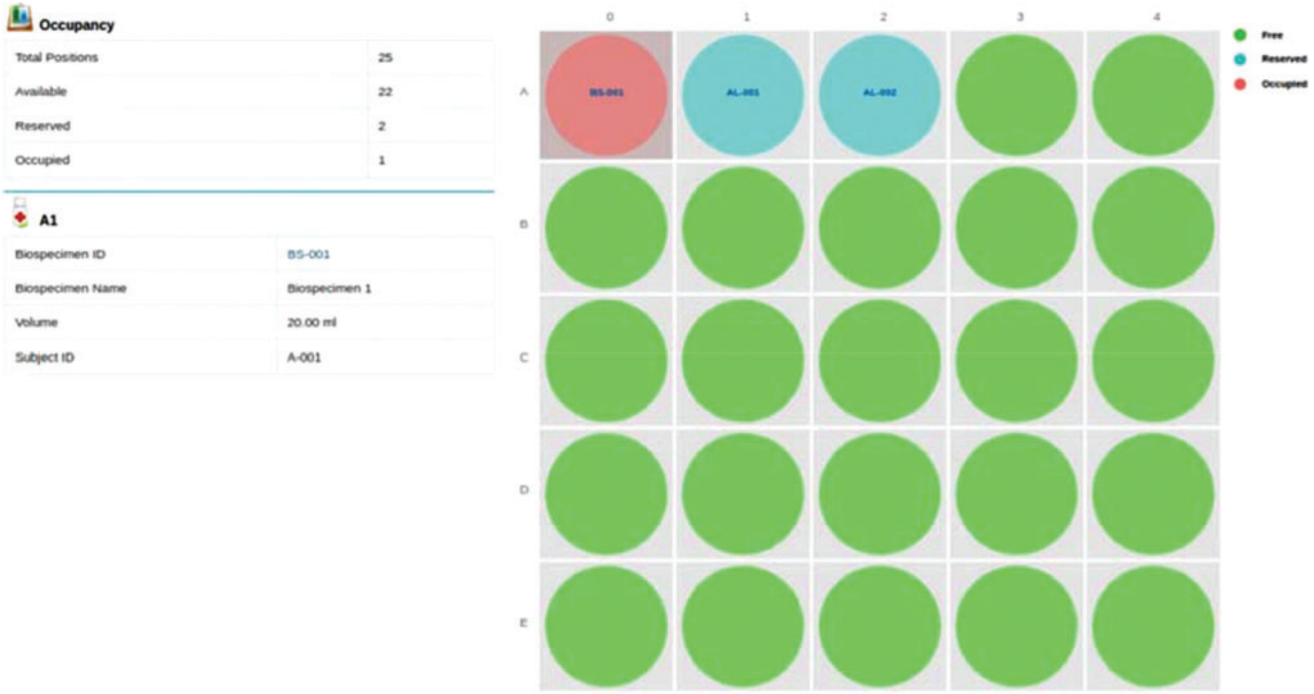

SUPPLEMENTARY FIG. S20. Graphical representation. The different storage positions for biospecimens or aliquots are graphically depicted in Supplementary Figure S21. Each circle represents an object position. A state of a position could be ‘‘free,’’ ‘‘reserved,’’ or ‘‘occupied.’’ A color with a different color represents each state: free = green; reserved = blue, and occupied = red.

Sample storage workflow

The following workflow was implemented to keep track of the storage position’s status:

First, the position created will have ‘‘Free’’ state. When creating a sample, if a position is defined, this position will change state to ‘‘Reserved.’’ Now if the sample is physically stored, then position’s state will change automatically to ‘‘Occupied’’ (Supplementary Fig. S21).

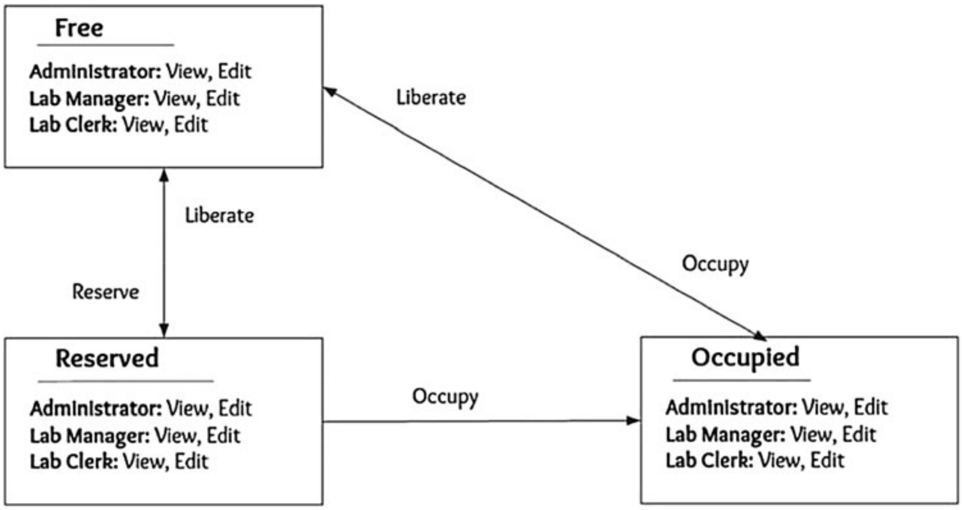

SUPPLEMENTARY FIG. S21. Storage location workflow.

Biospecimen Registration

The client returns the kits received from the biobank for collection, but this time with biospecimens inside. A biospecimen is a material taken from human body, such as tissue, blood, plasma, stool, and urine that can be used for diagnosis and analysis. A biobank staff member opens the kits and registers the biospecimen information into the system using the form shown in Supplementary Figure S22: title, id, type, volume, and storage location.

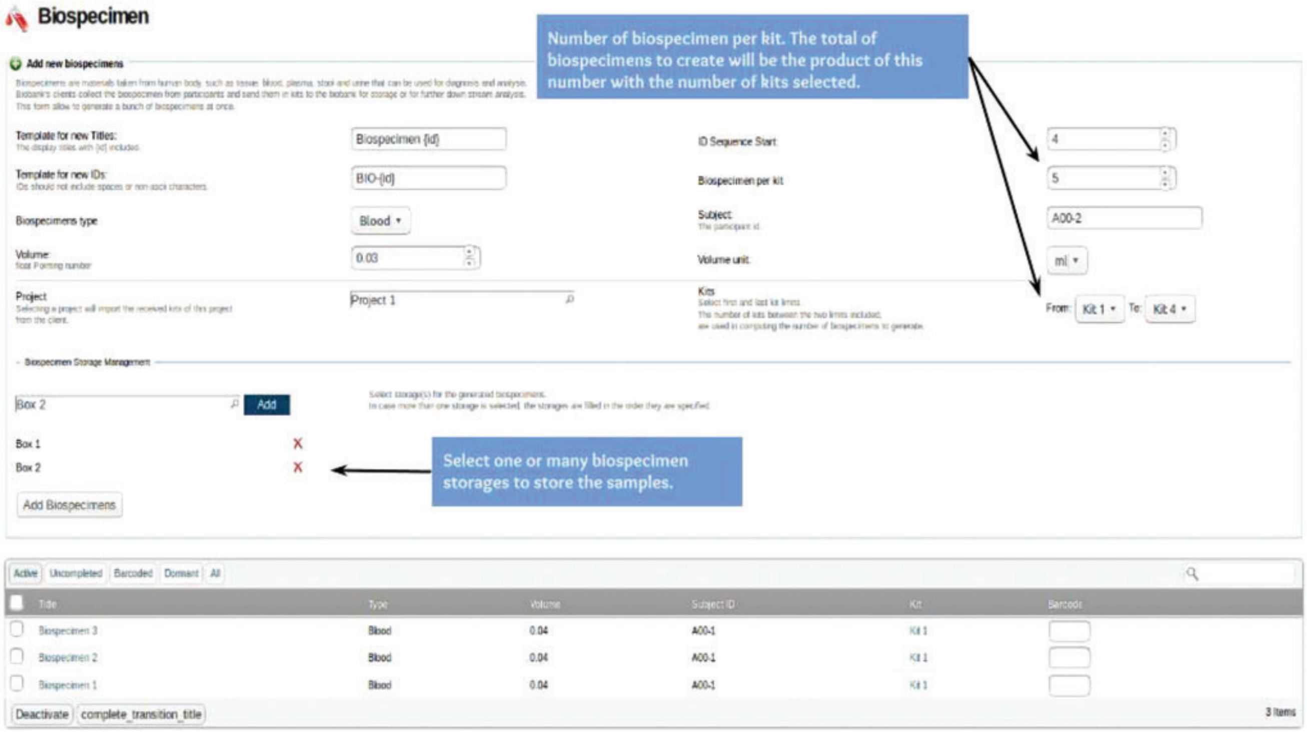

SUPPLEMENTARY FIG. S22. Biospecimen registration.

AR by Client

The client requests for an analysis to be carried out on specific biospecimens based on the case study of a particular project. The form used for creating AR is shown in Supplementary Figure S23. A biobank staff member selects the

biospecimen and the analysis services for use in downstream analyses. An analyst performs the predefined analyses physically in the laboratory using laboratory instruments and the results are then captured (Supplementary Fig. S24).

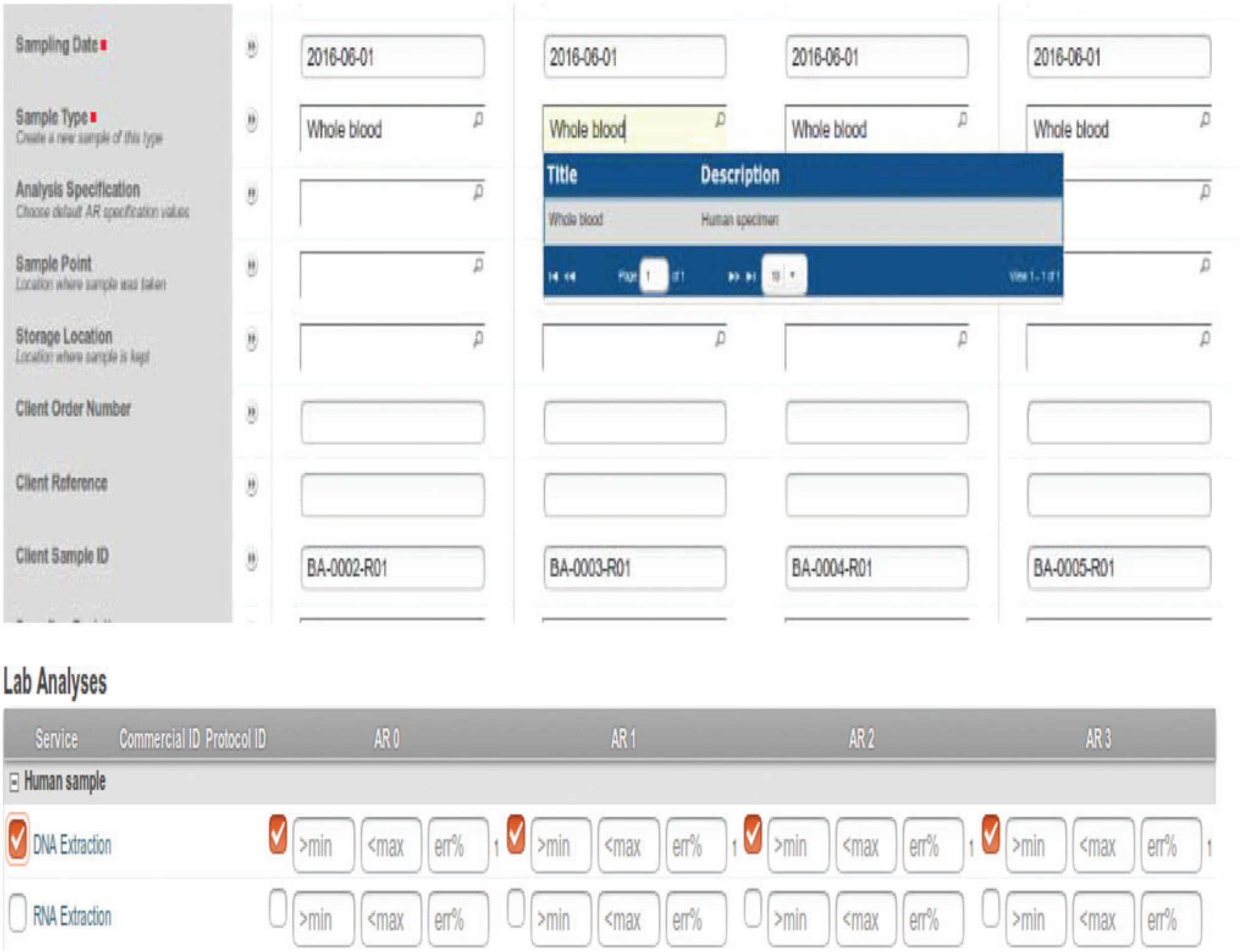

SUPPLEMENTARY FIG. S23. AR form, indicating the essential fields that must be completed by the clients requesting for analyses to be carried out on a human specimen.

Creating an instrument import interface for BioDrop μLITE and Qubit® 3.0 fluorometer

We identified two instruments that are key to human biobank and are lacking in BIKA LIMS, namely BioDrop μLITE and Qubit fluorometric instrument import interfaces for importing DNA/RNA analyses for utility in a biomedical laboratory. A template was created for BioDrop μLITE and Qubit fluorometric instrument interface for the analyses import form. This manages the submission of result files generated by instruments into the LIMS, which automatically import the data after upload to avoid any form of transcription error. BioDrop μLITE and Qubit fluorometric instrument result files are in comma-separated value format. The user can upload the generated instrument results files and import it into the LIMS by clicking on the submission button after uploading. This will significantly decrease the turnaround time and enhance accuracy of results (Supplementary Fig. S24).

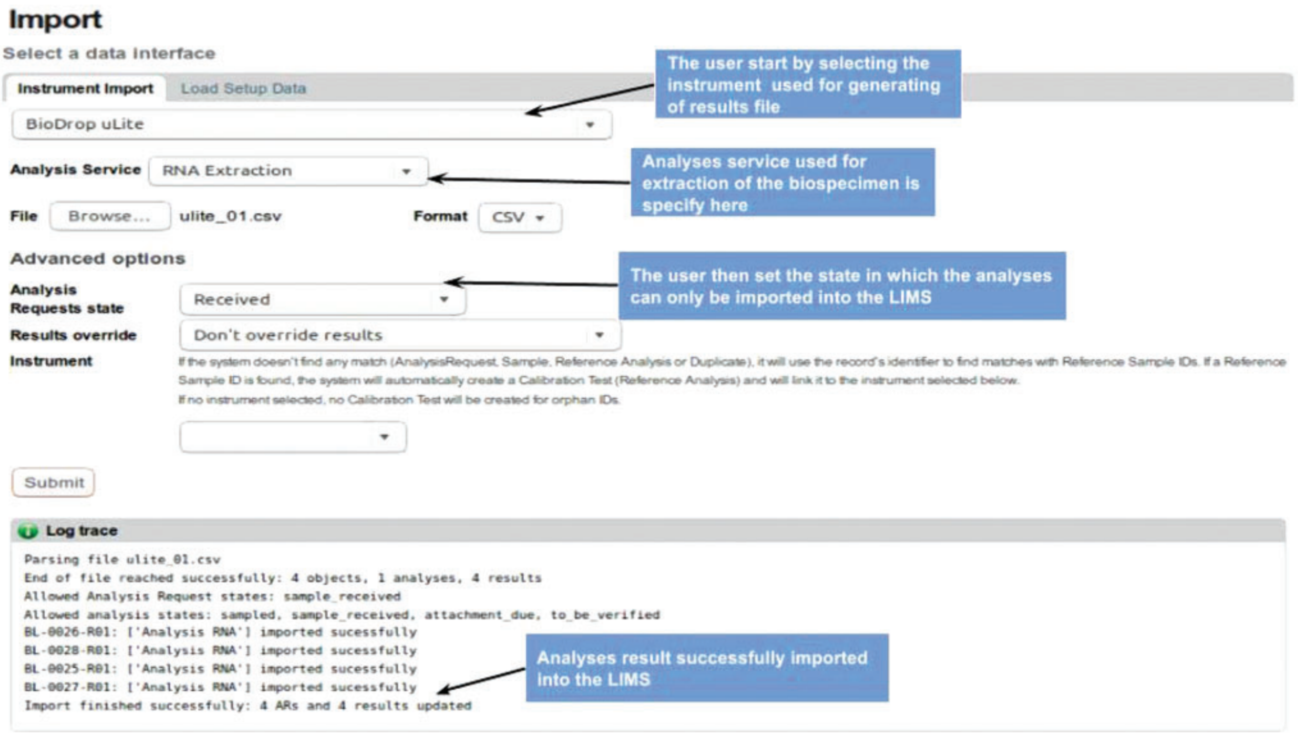

SUPPLEMENTARY FIG. S24. Selection of life technology instrument import interface and specifying the necessary analyses done, and uploading the required file to be imported into the LIMS. The results for DNA analysis successfully imported into the LIMS.
